# Leaving by staying: Social dispersal in giraffes

**DOI:** 10.1111/1365-2656.13582

**Published:** 2021-09-19

**Authors:** Monica L. Bond, Derek E. Lee, Arpat Ozgul, Damien R. Farine, Barbara König

**Affiliations:** ^1^ Department of Evolutionary Biology and Environmental Studies University of Zurich Zurich Switzerland; ^2^ Wild Nature Institute Concord NH USA; ^3^ Department of Biology Pennsylvania State University University Park PA USA; ^4^ Department of Collective Behavior Max Planck Institute of Animal Behavior Konstanz Germany

**Keywords:** anthropogenic effects, capture–mark–recapture, *Giraffa camelopardalis*, giraffe, net displacement, social network analysis

## Abstract

1. Dispersal is a critical process that shapes the structure of wild animal populations. In species that form multi‐level societies, natal dispersal might be social (associating with a different social community while remaining near the natal area), spatial (moving away from the natal area while continuing to associate with the same community) or both social and spatial (associating with a different community and moving away from the natal area).
2. For such species, classical spatial measures of dispersal, such as distance moved, might not capture social dispersal.
3. We examined dispersal outcomes for 67 male and 70 female giraffe calves over 7 years in a large, unfenced, ecologically heterogeneous landscape. We tested predictions about the influence of sex, food availability, low‐ and high‐impact human settlements, and local giraffe population density on social or spatial dispersal, dispersal distance, and age of dispersal.
4. We found that dispersal is sex‐specific, with females being predominately philopatric. When dispersing, both sexes did so at a mean of 4 years of age. Most (69% of total) young males dispersed, with 84% of male dispersers associating with a different adult female social community than that of their mother, but one in four of these dispersers remained spatially near to their natal area. For adolescent males that dispersed socially but not spatially, overlapping female social communities may represent a potential pool of unrelated mating partners without the risks of travelling to unfamiliar areas. Just 26% of young females dispersed and half of these continued to associate with the adult female social community into which they were born, confirming the importance of maintaining ties among females from calf to adulthood. Furthermore, individuals born farther from high‐impact human settlements were more likely to spatially or socially‐and‐spatially disperse, move greater distances from their natal areas, and disperse at a younger age.
5. Our study highlights the potential importance of social structure in dispersal decisions, and of tracking social structure when studying dispersal in multi‐level societies, as effective dispersal can be attained without large‐scale spatial displacements.

## Graphical abstract

This study is the first to investigate whether social dispersal occurs in animal societies characterised by open fission‐fusion group membership within stable multi‐group communities, where individuals can disperse with little to no spatial displacement.

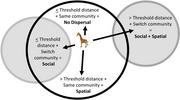

## Muhtasari

1. Mtawanyiko wa wanyama pori ni muhimu katika ustawi wao. Kwa spishi zinazoishi kwa makundi kama jamii, mtawanyiko unaweza kuwa wa kutoka katika kundi walikozaliwa kwenda katika makundi ya jirani katika eneo hilo au kuondoka kabisa toka katika makundi walikozaliwa na kuendelea kuungana na kundi la mwanzo.
2. Kwa spishi hizo kuelewa jinsi wanavyotumia maeneo yao kwa muda tofauti kwa kutumia njia kama umbali waliotembea inaweza kuwa na changamoto.
3. Tulichunguza taarifa za mtawanyiko wa twiga dume 67 na majike 70 ya watoto kwa miaka 7 kwa eneo kubwa la kiikolojia ambalo halina fensi. Tulijaribu kuthibitisha utabiri wa uwepo wa uhusiano kati ya jinsi ya twiga, uwepo wa chakula, usumbufu mdogo au mkubwa kutoka kwa makazi ya binadamu, wingi wa twiga na umri wao katika mtawanyiko.
4. Tulipata matokeo kuwa mtawanyiko unategemeana na jinsi ya twiga, majike hubaki pamoja lakini madume hutawanyika. Kwa wastani twiga jike au dume huanza kutawanyika wanapokuwa na umri wa miaka minne. Asilimia 69 ya twiga madume wadogo walitawanyika na asilimia 84 ya madume hao walikuwa wanachangamana na makundi mengine ya majike tofauti na yale ya mama zao. Kwa madume yaliyotawanyika kwenda kwenye makundi mengine karibu na walikozaliwa inaonesha muingiliano baina ya majike na madume yasiyokuwa na undugu bila kuhama. Asilimia 26 tu ya majike ndio waliweza kutawanyika lakini nusu yao waliendeleza mahusiano na makundi yao ya awali.
5. Utafiti wetu unaonesha umuhimu wa muundo wa jamii katika maamuzi ya kutawanyika/kuhama na kuendelea kufuatilia muundo wa jamii wakati tunatafiti mtawanyiko wa twiga katika ngazi zote za jamii.

## 1 INTRODUCTION

Dispersal is a critical component in animal population dynamics (Devillard & Bray, 2009). The natal dispersal process typically involves leaving the birth site or social group, making large displacements across unfamiliar habitat, and settling in a new home range or social group (Clobert et al., 2001; Matthysen, 2012; Wolff, 1994). While the benefits of dispersing include lower inbreeding probability, increased mating or social opportunities, and potentially lower resource competition in the new location (Greenwood, 1980; Schradin et al., 2012; Wolff, 1994), dispersers face increased predation risks and expend more energy when making large displacements (Clobert et al., 2001). These costs should drive individuals to express cost‐mitigating dispersal strategies (Klarevas‐Irby et al., 2021). One such strategy could be to disperse to a new social environment without making large spatial displacements.

In a number of social species, individuals live in stable social units or groups that fission–fusion with other units to form discrete higher‐level communities (Kummer, 1971; Papageorgiou et al., 2019). Several such communities can overlap in space, with members of different communities using the same areas but rarely or never being seen together (e.g. Bond et al., 2020; Bond, König, et al., 2021). These overlapping communities potentially provide dispersers with an opportunity to switch between communities without having to make large spatial displacements, thereby reducing the costs of dispersing while still allowing them to change associates: in such cases, spatial measures alone might provide an incomplete picture of dispersal. While dispersal between neighbouring groups has been described in many species, for example females of harem‐forming equids (Linklater & Cameron, 2009) and males of some primate societies (Cheney & Seyfarth, 1983), all these species exhibit tight and highly stable group membership. It remains to be investigated whether social dispersal occurs in societies characterised by more open fission–fusion group membership within stable multi‐group communities, where individuals can disperse with little to no spatial displacement.

Here we examined patterns of natal dispersal in a metapopulation of wild Masai giraffes (*Giraffa camelopardalis tippelskirchi*) inhabiting 2,200 km^2^ of an environmentally heterogeneous, unfenced landscape in northern Tanzania (Lee & Bolger, 2017). Giraffes are long‐lived (~25 year), large (800–1,200 kg), browsing ruminant ungulates inhabiting savanna woodlands in sub‐Saharan Africa (Dagg, 2014). Adult females in our study area form discrete but spatially overlapping social communities, comprised of approximately 60‒90 individuals (Bond, König, et al., 2021) with significant preferred and avoided associations towards other females (Bond et al., 2020) likely maintained over many years (e.g. Carter, Seddon, et al., 2013). Female giraffes maintain these long‐term relationships despite constant turnover (i.e. fission–fusion) in group membership (Bond et al., 2019; Innis, 1958; Leuthold, 1979), with these social preferences underlying community structure. Prehn et al. (2019) showed stable associations among a small population of adult male giraffes during several years, but over longer time periods and in larger populations associations between males appear to be less enduring than those of females (Carter, Seddon, et al., 2013), and older males are mostly solitary as they move among female groups (Bercovitch et al., 2006; Castles et al., 2019; Pratt & Anderson, 1982, 1985; VanderWaal et al., 2014). Giraffes thus exhibit a dynamic but ultimately structured social system characterised by multiple levels of organization, with the upper level comprising stable adult female communities that largely overlap in space (Bond, König, et al., 2021), potentially enabling young giraffes to disperse exclusively socially.

We then tested a set of literature‐derived predictions about factors influencing dispersal behaviour. First, we confirmed whether (P1), like other polygynous mammals (Clutton‐Brock & Lukas, 2012; Dobson, 1982; Greenwood, 1980; Wolff, 1994), giraffes exhibit male‐biased dispersal (female‐biased philopatry). The importance of social bonds among female mammals might mediate dispersal differences between sexes, as stable long‐term associations among adult females are common in social mammals (e.g. chimpanzees *Pan troglodytes* and gorillas *Gorilla gorilla*, Maryanski, 1987; chacma baboons *Papio hamadryas*, Silk et al., 2010; Bechstein's bats *Myotis bechsteinii*, Kerth et al., 2011; feral horses *Equus ferus caballus*, Marjamäki et al., 2013; giraffes, Carter, Seddon, et al., 2013; house mice *Mus musculus domesticus*, Evans et al., 2020). However, young females might occasionally benefit from leaving their natal areas, for example to reduce local competition for food resources (Schradin et al., 2012; Teichroeb et al., 2009). Thus, we predicted both male and female giraffes that dispersed would make large spatial displacements from their natal area but dispersing females would be more likely to nevertheless remain within their natal community (spatial, but not social dispersal; P2). We also tested whether (P3) dispersal probability is lower, dispersal distances shorter and dispersal age higher for calves born into communities with more preferred forage plants (Levi et al., in press), which indicates better habitat quality (Pettorelli et al., 2003). As humans can influence habitat value because of habitat fragmentation, poaching risk and behavioural disruption (Bond et al., 2019, 2020; Knüsel et al., 2019; Singh et al., 2012), we tested whether (P4) dispersal probability is higher, dispersal distances longer and dispersal age lower in giraffe calves born closer to human settlements. Finally, local competition is known to be a major driver of dispersal (positive density‐dependent dispersal; Loe et al., 2009; Marjamäki et al., 2013; Matthysen, 2005), so we tested whether (P5) spatial dispersal probability is higher for calves born in communities with higher local giraffe population density. We did not include predation risk as a factor to influence dispersal because lion predation on sub‐adult and adult giraffes is extremely rare in our study area (B. Kissui, pers. comm.).

## 2 MATERIALS AND METHODS

### 2.1 Study area

The Tarangire Ecosystem in northern Tanzania is a heterogeneous landscape with spatially varying vegetation and anthropogenic pressures on wildlife, ranging from habitats deep within protected national parks to habitats in close proximity to towns (dense concentrations of concrete structures surrounded by farms) and traditional homesteads of indigenous people, called bomas (scattered family compounds with several huts constructed from natural materials and surrounded by savanna rangeland). Bomas in our study area are occupied by pastoralist Masai people who typically do not poach giraffes for meat but may kill lions and other carnivores to protect livestock (Kissui, 2008). Towns are rare, are surrounded by farmlands and are inhabited by bushmeat poachers who often target giraffes (Kiffner et al., 2015). Further information about the study area is shown in Appendix 1 and Figure S1.

### 2.2 Field data collection

We conducted 42 independent daytime, fixed‐route transect driving surveys to photograph giraffes between January 2012 and October 2018. The Tarangire Ecosystem experiences three precipitation seasons per year (short rains [October–January]: 63 mm/month; long rains [February–May]: 100 mm/month; and dry [June–September]: 1 mm/month; Foley & Faust, 2010). We surveyed for giraffes near the end of each precipitation season following a robust design (Pollack, 1982) with each primary sampling period composed of two independent, consecutive secondary samples during which we drove all transects (dirt roads) in the study area. We sampled transects between 06:45 and 17:30. Transect density throughout the study area was high (0.42 km/km^2^) relative to adult female giraffe home range size (115 km^2^; Knüsel et al., 2019). All surveys included the same two dedicated observers (MLB and DEL), and we maintained a steady driving speed between 15 and 20 kph on all transects.

We identified each giraffe from its unique and unchanging coat pattern (Foster, 1966). When we encountered giraffes, we drove to within 150 m distance and photographed all individuals. We recorded the GPS location of the group, and distance from the camera to each giraffe (in meters) using a laser rangefinder (Bushnell Arc 1000; Overland Park, KS). We assessed a suite of physical characteristics, including body shape, relative length of the neck and legs, ossicone characteristics, and visual estimation of height to categorise giraffes into males and females, and into three age classes in the field: calf (<1 year), sub‐adult (1–3 years) or adult (≥4 years). Female giraffes in the wild typically first reproduce at 5–6 years of age (Lee & Strauss, 2016) but females become sexually mature at about 3.5 years, and males at about 4.5 years (Dagg, 1971).

### 2.3 Characterising dispersal

We used the computer program Wild‐ID to match patterns from the photographs (Bolger et al., 2012). Photographic records were converted into individual encounter histories and social networks (Farine & Whitehead, 2015) from which we derived adult female social communities (Appendix 2; Figure S1a).

For our dataset of potential dispersers, we ensured that each individual was first sighted as a calf and that we had resight data for the individual over at least 5 years from the first sighting, representing a sufficient time period to detect natal dispersal. Thus, our dataset included all individuals that were (a) known to be calves in 2012 or 2013 (the first 2 years of our 7‐year study) and (b) detected at least once in each of the following intervals: 2012–2014, 2013–2015, 2014–2016, 2015–2017 and 2016–2018. This resulted in a subset of 137 potential dispersers (67 M and 70 F, herein calves) from a total of 328 calves born in 2012 and 2013. The number of detections per calf ranged from a minimum of 6 to a maximum of 29 (Figure S2). The subset included calves born in every part of the study area (see Figure S1b).

**FIGURE 1:**
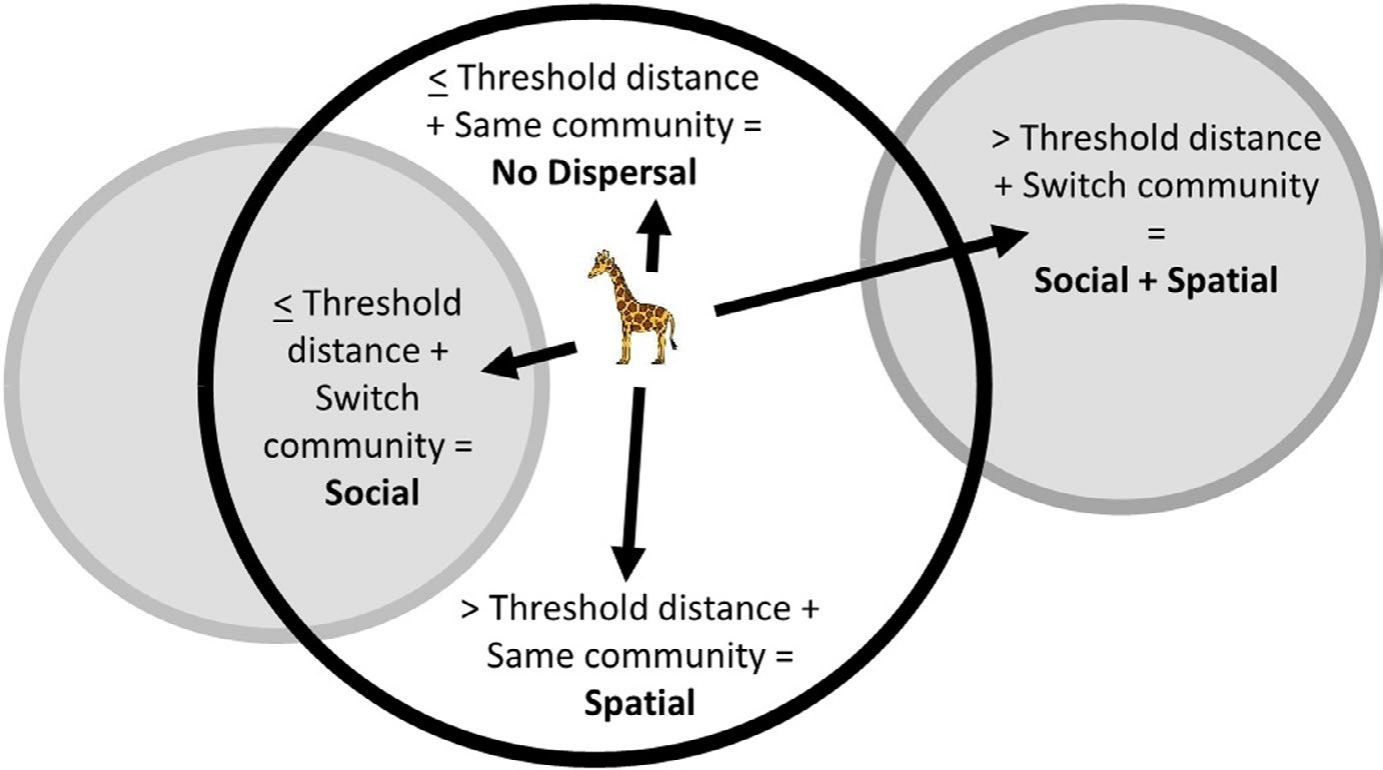
Types of dispersal of a hypothetical juvenile giraffe in the Tarangire Ecosystem. Black circle denotes social community of its mother, and grey circles denote social communities different from its mother. Arrows indicate spatial distance travelled. Social dispersal is joining a different social community with dispersal distance ≤ radius of mean adult female home range in its natal community, from first detection as a calf. Spatial dispersal is dispersal distance > radius threshold while remaining in the natal social community. Social‐and‐spatial dispersal is dispersal distance > radius threshold and joining a different social community. No dispersal is dispersal distance ≤ radius threshold and remaining in the natal community

We next quantified which calves were philopatric and which dispersed. Giraffe calves cannot be assigned to a mother unless extended suckling is observed, which was rarely observed with our study design, so we could not define dispersal based on proximity to mother. Instead, we classified individuals into four distinct dispersal types based on social and spatial movements: (a) non‐dispersers; (b) social dispersers; (c) spatial dispersers and (d) social‐and‐spatial dispersers (Figure 1), according to the following criteria:

#### 2.3.1 Social dispersal

Social dispersal occurs when a giraffe associates with an adult female community that is different from its natal community. We created a social network of adult females and used an algorithm to identify distinct communities using packages *asnipe* (Farine, 2013), *igraph* (Csárdi & Nepusz, 2006) and *assortnet* (Farine, 2014) in R version 3.5.3 (R Core Development Team, 2019; see Appendix 2 for methods). We assigned each detection for each calf to an adult female community based on the community membership of the adult females in the group in which the calf was observed. We then quantified if and when the calf associated with a different community from the temporal patterns of its assignments into communities. In some cases, the calf appeared to switch communities several times. In these cases, we differentiated among the following situations: (a) when it was always associated with the natal community, social dispersal = 0; (b) when it was associated with a community that was adjacent to or overlapping the natal community but then was detected subsequently thereafter back in the natal community, social dispersal = 0 (we assume this represents exploratory social visits, similar to a spatial sortie described below); (c) when the calf shifted to, and was subsequently always associated with a community or communities different from the natal community, social dispersal = 1. Adult female social communities were highly stable over the 7‐year time frame (Appendix 2), so we were confident that calves permanently switching community associations after the maximum age of weaning (>18 months; Dagg & Foster, 1976) were doing so on their own and without their mother.

#### 2.3.2 Spatial dispersal

To investigate spatial dispersal, we calculated net displacement: the Euclidean distance between the first detection location of each calf and each successive location it was observed (Turchin, 1998). We considered the distance from the first sighting to its final sighting to be the dispersal distance. As parental care is provided by female and not male giraffes, we defined spatial dispersal as a dispersal distance that was beyond the radius of the average adult female 95% kernel home range (Clobert et al., 2001; Gaillard et al., 2008; Loe et al., 2009), which we calculated separately for each community. Our study area size of 2,200 km^2^ represents approximately 19 non‐overlapping adult female home ranges based on the overall average of 115 km^2^ (Knüsel et al., 2019); therefore, we presumed our study area provided adequate space to detect a range of dispersal movements.

Some individuals may have travelled beyond the threshold distance and back again several times, and thus whether these individuals engaged in spatial dispersal is less clear. To confirm spatial dispersal, we fitted a smoothed line to the distance data for the subset of 53 calves whose final detection was beyond the threshold. We then calculated how many of the detections that occurred after the fitted line exceeded the threshold were beyond the threshold distance. If more than half of these detections were beyond the threshold, we classified the individual as a spatial disperser. This procedure removed four females from the spatial disperser category (they were re‐classified to non‐dispersers that made sorties). Figure S3 shows the data from these 53 individuals. For the other individuals, we quantified which made sorties away from the natal location (exceeding the threshold distance in one observation) but then returned.

#### 2.3.3 Dispersal age

By calculating distance from natal location for every detection rather than simply the final detection, we could estimate the age at which the individual was first recorded beyond the threshold distance, and the age at which it remained spatially dispersed. We used photogrammetry to estimate age from heights (Appendix 3) and assigned each calf an age in months for each detection. We attributed the age of social dispersal as its first detection with a community different from the natal community, and the age of spatial dispersal as its first detection beyond the threshold distance described above. We also calculated age of first sortie, and age that an individual was first seen in a bachelor herd, which is defined as a group comprising majority of males. Females are often found in bachelor herds (VanderWaal et al., 2014).

### 2.4 Data analysis

#### 2.4.1 Dispersal patterns

We first tested for sex differences in dispersal propensity (P1) and examined dispersal type (any form of social or any form of spatial dispersal), given that the animal dispersed (P2), with generalized linear mixed models using a logistic regression with a binomial error distribution and natal community as a random effect. To test whether males were more likely to disperse (P1), we created a model in which the response variable captured whether any dispersal was detected and fitted sex as the predictor. To test whether dispersing females were less likely to switch to a different community than dispersing males (P2), we created a model in which the response variable captured whether the disperser exhibited any form of social dispersal and fitted sex as a predictor. We also tested whether males and females were equally likely to make large spatial displacements (P2) by fitting a model in which the response variable captured whether the disperser exhibited any form of spatial dispersal and fitted sex as a predictor. Finally, we tested for differences in age at first sortie, and age first seen in a bachelor herd, between males and females using Welch's two‐sample *t* tests.

#### 2.4.2 Correlates of dispersal type, distance and age

Second, we were interested in how socio‐ecological factors might predict the dispersal characteristics of young giraffes, including dispersal type (no dispersal, social, spatial, social‐and‐spatial), distance moved from first detection as a calf and dispersal age. We tested the effects of following covariates of the natal community: proportion of area with >10% cover of three preferred giraffe forage species (*Vachellia tortilis*, *V. drepanolobium* and *Dichrostachys cinerea* [P3]), distances to low‐ and high‐impact human settlements (P4), and local giraffe population density (all adults detected within a community's home range regardless of community membership [P5]). See Appendix 4 for methods of calculating covariates. We developed and ran 20 a priori regression models—including a null model—with single and various additive and interactive combinations of the socio‐ecological covariates as well as sex. We ran the same models in three analyses: dispersal type, dispersal distance and dispersal age. We did not include two highly correlated variables together in any models (correlation coefficient >0.50; Table S1).

1. Dispersal type: To characterise dispersal probability by type as a function of the covariates, we assigned each individual one of four distinct dispersal types as the response variable: no dispersal, social, spatial or social‐and‐spatial dispersal. We then fit a multinomial model with the four response variables and ‘no dispersal’ as the reference level using r package nnet (Venables & Ripley, 2002). We calculated sex‐specific predicted probabilities of dispersal type associated with the covariates from the top‐ranked models using r package MNLpred (Neumann, 2021).
2. Dispersal distance: We modelled, for all animals, dispersal distance from origin (km) as a function of the covariates using linear regression and a Gaussian error distribution.
3. Dispersal age: For animals that dispersed, we modelled approximate dispersal age (in months) as a function of the covariates, using linear regression and a Gaussian error distribution.

**TABLE 1:** Summary statistics of number of male (M: total of 67) and female (F: total of 70) giraffe calves in each dispersal class (proportion within sex), mean final distance from origin (km), and maximum movement distance from origin (km)

	No dispersal	Social dispersal	Spatial dispersal	Social‐and‐spatial dispersal
Sex				
F	52 (0.74)	4 (0.06)	9 (0.13)	5 (0.07)
M	21 (0.31)	12 (0.18)	8 (0.12)	26 (0.39)
Mean final movement distance (km)				
F	2.95	4.43	9.63	16.18
M	3.23	3.95	13.04	17.39
Maximum movement distance (km)				
F	5.80	5.86	16.29	26.14
M	6.90	6.56	19.21	32.85

We applied an information‐theoretic approach to model selection (Burnham & Anderson, 2002), using Akaike's information criterion corrected for small samples (AIC_c_) to compare and rank models, and AIC_c_ weights to represent the relative likelihood of a model.

## 3 RESULTS

The community detection algorithm parsed adult females into 12 social communities (Appendix 2; Figure S1a). The threshold radius distance for spatial dispersal varied by adult female social community and ranged from 4.95 to 7.77 km.

### 3.1 Dispersal patterns

Males had a significantly greater propensity than females to disperse (*ß*_SexM_ = 1.85 ± 0.41, 95% CI = 1.12–2.70, *p* < 0.001; random effect standard deviation = 0.03); 26% (*n* = 18) of females and 69% (*n* = 46) of males dispersed (Table 1; Figure S4). Of animals that did disperse, males were significantly more likely to switch to a new community (*ß*_SexM_ = 1.53 ± 0.64, 95% CI = 0.37–2.79, *p* = 0.02; random effect standard deviation = 0.55), whereas males and females were equally likely to make a large spatial displacement (*ß*_SexM_ = −0.20 ± 0.68, 95% CI = −1.61 to 1.13, *p* = 0.77; random effect standard deviation = 0.27). The effect of community membership (the estimated among‐community standard deviation from the random effect) was much lower in magnitude than the contribution of sex on the propensity to disperse and the probability to express social dispersal, suggesting there is relatively little variation among communities.

Females conducted their first spatial sortie at a significantly younger age than males (mean female sortie age = 17 months, *SD* = 12, range 2‒50 and mean male = 33 months, *SD* = 15, range = 4‒72; *t* = −6.15, *df* = 91, *p* < 0.001; Table 2). We suspect long distances moved when the calf was <8 months of age was a sortie with its mother because giraffe calves wean between 8 and 18 months of age (Dagg & Foster, 1976); such sorties included six females (12%) and two males (4%). Two females (4%) and eight males (16%) conducted a first sortie after the mean spatial dispersal age for their respective sex, so it is possible that some calves we categorised as spatial dispersers might return to near their natal site. We did not detect a difference between the sexes in age first seen in a bachelor herd (*t* = −0.23, *df* = 12, *p* = 0.82; Table 2).

**TABLE 2:** Mean age in months (standard deviation) of first sortie (movement > the radius of an adult female home range, but later return to within the threshold distance), age first detected in a bachelor herd (group comprised of majority males), age of social dispersal and age of spatial dispersal for juvenile giraffes in the Tarangire Ecosystem, Tanzania, 2012‒2018. F, female; M, male

Sex	Mean first sortie age	Mean first bachelor herd	Mean social dispersal age	Mean spatial dispersal age
F	17 (12) *n* = 50 range 2‒50	39 (15) *n* = 8 range 26‒66	52 (19) *n* = 9 range 22‒73	43 (22) *n* = 14 range 16‒78
M	33 (15) *n* = 51 range 4‒72	40 (18) *n* = 40 range 6‒88	45 (18) *n* = 38 range 12‒74	46 (16) *n* = 34 range 19‒78

### 3.2 Correlates of dispersal type, distance and age

#### 3.2.1 Dispersal type

**FIGURE 2:**
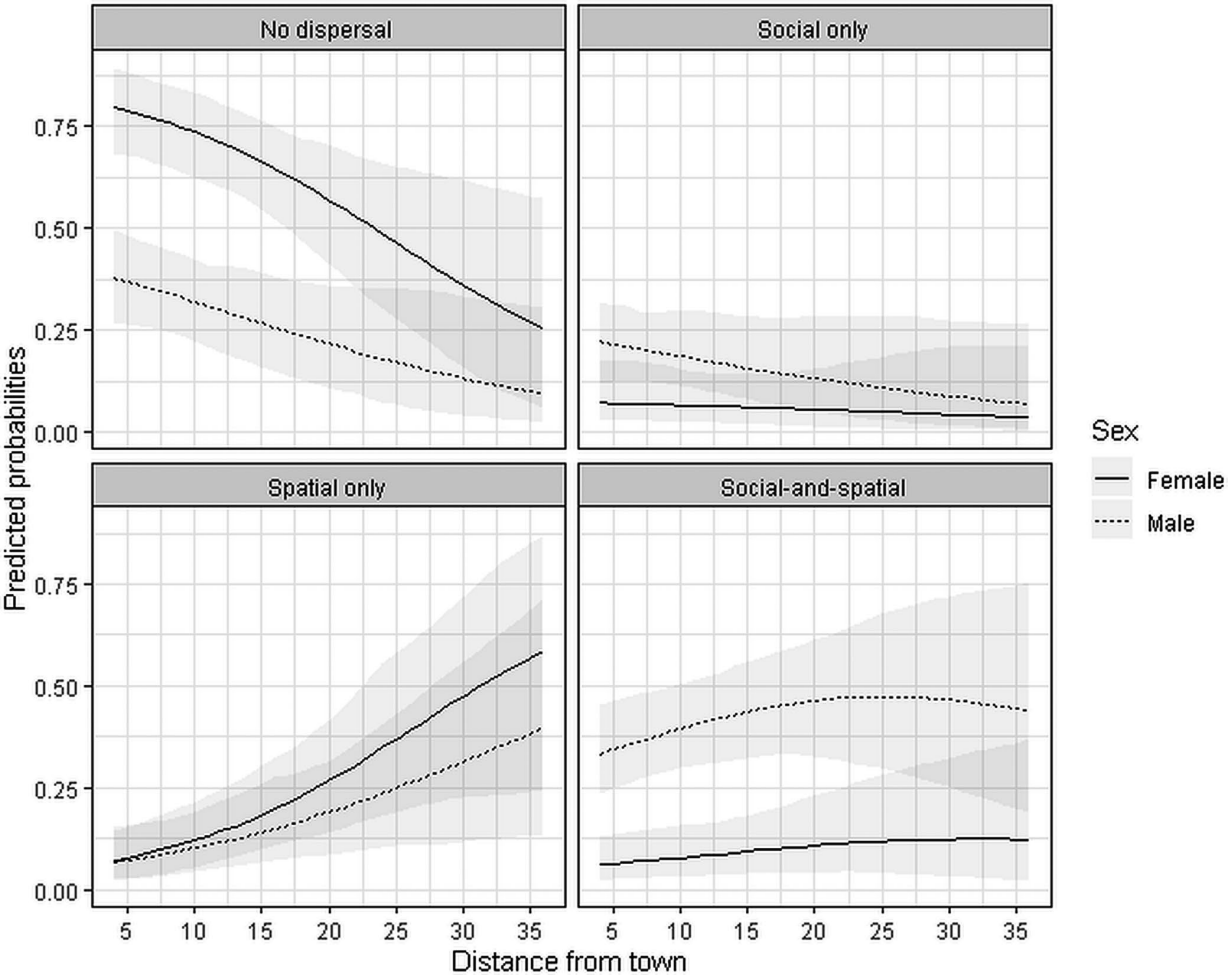
Mean predicted probabilities of dispersal type for 137 male and female Masai giraffe calves born into social communities at different distances from a town (km) in the Tarangire Ecosystem, Tanzania, from 2012 to 2018. Coefficients from top‐ranked model

The top‐ranked dispersal type model included sex and distance to town (Table S2). The probability of being a social disperser or a social‐and‐spatial disperser versus not dispersing was significantly higher for males than females (Table 3; Figure 2a). Relative risk ratios (exponents of the coefficients) indicate that males were more than seven times more likely to socially disperse, and 13 times more likely to socially‐and‐spatially disperse than females, compared to not dispersing. Distance to high‐impact human settlements (towns) was positively correlated with probability of dispersing spatially and socially‐and‐spatially versus not dispersing (Table 3). The risk ratios suggest that for every kilometre a calf's natal community was located from a town, the probability of spatially dispersing increased by 0.01 and the probability of socially‐and‐spatially dispersing increased by 0.07.

**TABLE 3:** Coefficient estimates, standard errors (*SE*), *z*‐scores or *t* values, and *p* values from regression models explaining variation in dispersal type (reference level = no dispersal), distance and age as functions of socio‐ecological covariates from top‐ranked models, for male and female giraffe calves in the Tarangire Ecosystem of northern Tanzania, 2012‒2018. SexM is male, and DistTown is mean distance (km) from all locations of the giraffe's natal community members to the nearest high‐impact human settlement

Dispersal type												
All giraffes	Intercept coefficient	*SE*	*z* score	Pr(>|*z*|)	SexM coefficient	*SE*	*z* score	Pr(>|*z*|)	DistTown coefficient	*SE*	*z* score	Pr(>|*z*|)
Social only	−2.62	0.65	−4.05	<0.001	2.01	0.63	3.17	<0.001	0.01	0.04	0.14	0.89
Spatial only	−3.06	0.58	−5.32	<0.001	0.74	0.59	1.24	0.21	0.11	0.03	3.47	<0.001
Social‐and‐spatial	−3.01	0.58	−5.14	<0.001	2.56	0.56	4.56	<0.001	0.07	0.03	2.12	0.03

Coefficients	Estimate	*SE*	*t* value	Pr(>|*t*|)
Dispersal distance				
(Intercept)	2.73	0.99	2.77	0.01
SexM	3.44	1.08	3.19	<0.001
DistTown	0.31	0.07	4.76	<0.001
Dispersal age				
(Intercept)	52.67	4.26	12.35	<0.001
DistTown	−0.55	0.25	−2.19	0.03

#### 3.2.2 Dispersal distance

The top‐ranked dispersal distance model (Table S2) indicated that males moved greater distances than females (Figure S5) and distance of natal area to towns was positively correlated with dispersal distance, meaning the farther an individual was born from a town, the farther away it moved from its origin (Table 3). The top model explained 20% of the variation in dispersal distance (adjusted *R* ^2^ = 0.198) and carried 86% of the weight in our candidate model set (Table S2). No other variables were correlated with dispersal distance. A post‐hoc Wilcoxon rank‐sum test comparing our subsample of calves (*n* = 137) with the other calves born during the first two years of the study that we did not include due to lower numbers of detections (*n* = 191) indicated natal distances from towns did not differ between these groups (*W* = 14,008, *p* = 0.28; Figure S6), meaning that our subsample of calves was not biased with respect to distance from towns.

#### 3.2.3 Dispersal age

Most males and females that dispersed, either socially or spatially, did so between about 43 and 52 months, or approximately 4 years of age (Table 2). We detected 38 of the 47 social dispersers ≥1 additional time after they were observed with non‐natal communities, but 9 individuals were seen only once in a non‐natal community with no detections thereafter. Hence, we also could not rule out that those individuals were socially exploring at an older age and might eventually return to their natal community.

The best model explaining dispersal age included a negative correlation with distance of natal area to town (Table 3; Table S2), suggesting that the farther an individual was born from a town, the younger it dispersed, with no significant effect of sex. However, the top model explained only 9% of the variation in dispersal age (adjusted *R* ^2^ = 0.091).

## 4 DISCUSSION

Young male giraffes were overall more likely to disperse, and to disperse greater distances than females, in agreement with our predictions (P1). Male dispersers were more likely than females to switch social communities, also consistent with our expectations (P2), but 26% of males and 22% females did so without expressing significant spatial displacement, remaining near their natal site yet associated with a different adult female community from the one into which they were born (social dispersal). Thus, our results provided mixed support for sex differences in social dispersal. Dispersing males and females were equally likely to make large spatial displacements (P2), and both sexes dispersed at approximately 4 years of age. Contrary to our predictions, calves born closer to a town (P4) were less likely to disperse spatially or socially‐and‐spatially, dispersed shorter distances and dispersed at an older age. Neither food availability (P3) nor local giraffe population density (P5) influenced dispersal type, distance moved or age.

### 4.1 Sex‐biased dispersal and mating strategy

Not surprisingly, most young female giraffes remained ‘at home’ (i.e. did not disperse). For polygynous mammals such as giraffes in which males do not defend territories, Greenwood (1980) suggested females would benefit most from philopatry. Both sexes, however, benefit from knowledge about local resources, so male‐biased dispersal in giraffes is likely to be related to the mating system and breeding tenures of males (Wolff, 1994).

Little is known about mating strategies of giraffes in the wild, as paternity is not directly observable. It is well established that young males form bachelor herds, becoming more solitary as they age and seek females in oestrous (Bercovitch et al., 2006; Castles et al., 2019; Pratt & Anderson, 1982, 1985; VanderWaal et al., 2014), but it is not known which males are the most successful breeders, or for how long they remain as breeders. Wolff (1994) postulated that female philopatry in polygynous mammals is associated with the length of breeding tenure of the opposite‐sex parent. Intense competition for dominance often results in short breeding tenures of adult males (e.g. red deer *Cervus elaphus*, Clutton‐Brock et al., 1982; northern elephant seals *Mirounga angustirostris*, Le Boeuf, 1974), resulting in daughters reaching sexual maturity after their father's tenure has ended. On the other hand, mothers are more likely to be present when their sons reach reproductive age. Consequently, sons must disperse to avoid their mothers, whereas daughters can be philopatric if their fathers are absent. Giraffe gestation is approximately 15 months and male giraffes have a life span upwards of 20 years (Berry & Bercovitch, 2012), so in order for daughters to reach sexual maturity (~4 years) in the absence of their fathers, the optimal breeding tenure of adult males that minimises the likelihood of incest is <5 years (assuming inbreeding has fitness costs). Therefore, successful reproduction is probably restricted to the oldest sexually active bulls (senescence in male giraffes is not known). Studies of dispersal such as ours, combined with data on longevity, can provide important insights into the mating strategies of animals. In addition, future studies using techniques such as net squared displacement modelling could yield further insights into the different movement modes of individual giraffes (Börger & Fryxell, 2012).

### 4.2 Female philopatry and social bonds

Our observed philopatry by most females, together with evidence of a structured female social system (Bond et al., 2020; Bond, König, et al., 2021; Carter, Brand, et al., 2013; Carter, Seddon, et al., 2013; VanderWaal et al., 2014), suggests that it is important for female giraffes to maintain social ties and be connected with many members of the community throughout their lives (Bond, Lee, Farine, et al., 2021), as evidenced in a variety of other taxa (e.g. chacma baboons, Silk et al., 2010; rock hyraxes *Procavia capensis*, Barocas et al., 2011; bighorn sheep *Ovis canadensis*, Vander Wal et al., 2015; blue monkeys *Cercopithecus mitis stuhlmanni*, Thompson & Cords, 2018; rhesus macaques *Macaca mulatta*, Ellis et al., 2019). Female giraffes might benefit from social bonds through sharing in protection of offspring, with calves forming crèches accompanied by one or a few females so that mothers can range relatively far from their calves to drink or forage (Dagg & Foster, 1976; Leuthold, 1979; Young & Isbell, 1991). Although rare in the wild, giraffes also have been observed to allow allonursing (Bond & Lee, 2019). As female giraffes tend to associate preferentially with related females (Bercovitch & Berry, 2012; Carter, Seddon, et al., 2013), cooperative care of offspring may impart inclusive fitness benefits, thus lowering dispersal propensities and leading to the formation of female‐based kin societies (Bowler & Benton, 2005). Therefore, the advantages of associating with familiar, and likely related, individuals may mediate social philopatry in female giraffes.

We had predicted that the infrequent instances of female dispersal would be spatial, yet 22% of the 18 female dispersers still dispersed socially and 28% dispersed socially‐and‐spatially. What factors might impel a young female to disperse away from the community in which she was born and raised? We found no evidence for an influence of food availability or population density, but sociability may play a role in dispersal decisions. Long‐term research on marmots *Marmota flaviventris* found female yearlings that interacted with fewer others and were less socially embedded were more likely to disperse (Blumstein et al., 2009). Furthermore, when the young female showed more amicable behaviors towards its mother and other yearling females, dispersal was less likely (Armitage et al., 2011). More‐intensive sampling of female giraffe calves and sub‐adults to obtain estimates of sociability may similarly reveal that females are more likely to socially disperse if they are less integrated in their natal communities.

### 4.3 Male social dispersal

Most young male giraffes dispersed, but one in four of them (26%) did so by ‘leaving without going far away’, that is, to disperse socially without making large spatial displacements. Such social dispersal supports the notion that the social dynamics within and between discrete communities—which can overlap in space with other communities—underpin metapopulation structure. If adult female communities indeed are extended family groups, males may be able to seek unrelated mating partners and avoid inbreeding without the need to travel long distances into unfamiliar areas, simply by associating with a nearby social community different from the one into which they were born—and thereby continue to benefit from local knowledge about resources. The proximity of a nearby community enables an individual to make occasional social sorties prior to permanently dispersing, which may reduce social resistance to dispersal (Armansin et al., 2020). However, the long‐term genetic costs of persistent dispersal into overlapping or adjacent communities can only be evolutionarily stable if some individuals forego the benefits of this strategy and move into more distant communities (Cheney & Seyfarth, 1983). Male giraffes can live upwards of 20 years, and adult female home ranges are relatively large (~115 km^2^): if males must reside far enough from their female kin to minimise the risk of inbreeding, this would explain why most males both dispersed long distances and associated with different social communities.

The fact that one‐third of males did not disperse at all was surprising, but our sample of males likely represents a transition stage from calf to sub‐adult. Male giraffes can become sexually mature at approximately 4.5 years of age (Dagg, 1971), but likely do not successfully mate until much older in the wild, as demonstrated by age‐related increases in sexual activity and faecal androgen metabolites (Seeber et al., 2013). In contrast to mature dominant males, sub‐adult male giraffes (≤4 years) and younger mature bulls in a South African population had higher faecal glucocorticoid levels associated with ‘puberty’ (Wolf et al., 2018). High glucocorticoid levels suggested they might still be subordinate to the dominant bulls. Therefore, it is possible that with additional years of data, more of the young males in our study area that did not disperse within our time frame might eventually move away from females to whom they are related, once they can effectively challenge other bulls in the dominance hierarchy. In our 7‐year dataset, our sample of females became old enough to begin reproducing by the last time period in our study; thus, we are more confident our results represent females that have settled into their final breeding areas and social communities.

Our results differ markedly from the only other ungulate species where social‐and‐spatial dispersal has been examined, the feral horse (Cameron et al., 2009; Linklater & Cameron, 2009; Marjamäki et al., 2013). Like female giraffes, mares disperse from their natal groups coinciding with their sexual maturation, but groups into which they disperse are predicted by proximity to the natal group (Linklater & Cameron, 2009). Thus, mares appeared to exhibit predominately social dispersal. However, feral horses have tighter and more stable group membership than giraffes; thus, spatial without social dispersal is not possible. Furthermore, in social species with highly stable group membership, including feral horses, many primates (e.g. vervet monkeys *Cercopithecus aethiops*; Cheney & Seyfarth, 1983) and meerkats (*Suricata suricatta*; Cozzi et al., 2018), dispersers must navigate a relatively fixed ‘social landscape’, which can require avoiding existing groups to find space to form a new group or joining a pre‐existing social group. In contrast, giraffe groups merge and split constantly over the course of a single day (Innis, 1958; Leuthold, 1979), for example adult females are on average only seen with the same individual females in half all sightings at most (Bond, Lee, Farine, et al., 2021; Foster & Dagg, 1972). Thus, young giraffes are confronted with a different ‘social landscape’ relative to species that form stable groups. Our research adds a new layer of understanding to the patterns of social versus spatial dispersal in a fission–fusion species with stable adult female social communities (as opposed to stable groups).

### 4.4 Socio‐ecological correlates of dispersal

Contrary to our expectations, individuals born farther from towns, deep in the heart of the protected areas, were more likely to disperse spatially or socially‐and‐spatially, move greater distances from their natal area and disperse at a younger age. One possible explanation is that giraffes born closer to towns face novel threats such as farms, roads and dense human settlements which may obstruct dispersal movements (sensu Singh et al., 2012). Other explanations may be less available habitat, thereby constricting individuals into smaller areas, or better quality of habitat in areas closer to human settlements indicating higher carrying capacity. Matthysen (2012) reported that dispersers are more likely to leave habitats of lower quality caused by shortage of resources, high levels of parasitism or physical disturbance. The giraffe communities in the northern part of our study area are not only situated closer to towns but also encompass more volcanic soils within the home ranges (Bond, König, et al., 2021), which are particularly fertile and may support enhanced forage quality (Hansen et al., 1985). Indeed, distance to towns was negatively correlated with proportion of volcanic soils in our study area (Bond, König, et al., 2021). This higher‐quality vegetation might dissuade young giraffes from leaving these areas despite possible disturbance from human settlements.

We cannot exclude that the data in our current study might suffer from a spatial sampling bias, whereby some animals near the edge of our study area—closer to towns—were more likely to disperse beyond our survey boundaries, although suitable habitat outside protected areas is sparse around towns. Our study area contains most of the suitable giraffe habitat in the region; thus, we expected most animals to remain within our survey boundaries, but dispersal into outlying areas potentially could occur. Conducting additional surveys to identify giraffes in these outlying areas could find more longer‐distance dispersers originating from areas near towns.

## 5 CONCLUSIONS

Natal dispersal patterns of giraffes are virtually unknown, and quantifying dispersal is an important step towards understanding the fine‐scale spatial demographic dynamics for giraffes as well as other herbivores with similar multi‐level, fission–fusion social systems. Our dataset, capturing multiple years of observations across a large metapopulation of giraffes, represents a unique opportunity to investigate social versus spatial dispersal, and elucidate socio‐ecological factors that mediate dispersal patterns.

Clearly, spatial measures alone may not provide a complete picture of dispersal for social species. We show that most young female giraffes tend to continue associating with their natal social community, whereas males tend to associate with female social communities different from their mother's, often even without moving long distances, with anthropogenic factors influencing dispersal behaviour. Understanding the patterns and drivers of the dispersal process is key for predicting how dispersal influences metapopulation dynamics, habitat colonization and gene flow, and is particularly important for conservation of endangered species such as giraffes (Bolger et al., 2019).

## CONFLICT OF INTEREST

All authors declare no conflict of interest.

## AUTHORS' CONTRIBUTIONS

M.L.B., D.E.L., A.O., B.K. and D.R.F. conceived the ideas and designed the methodology; M.L.B. and D.E.L. collected the data; M.L.B. and D.R.F. analysed the data; M.L.B. led the writing of the manuscript. All authors contributed critically to the drafts and gave final approval for publication.

## Supporting information

Supplementary Material

## Data Availability

Dataset and R code used to quantify spatial dispersal are openly available on figshare https://doi.org/10.6084/m9.figshare.15104115 (Bond, Lee, Ozgul, et al., 2021).
