# Supplementary material for: Leaving by staying: Social dispersal in giraffes
Source: J Anim Ecol. 2021 Sep 19;90(12):2755–66. doi: 10.1111/1365-2656.13582 (PMC9291750; doi:10.1111/1365-2656.13582)
Supplement: Supplementary file 1 — Supplementary Material [file JANE-90-2755-s001.pdf]

Leaving by staying: Social dispersal in giraffes

M. L. Bond[1,2,*], D. E. Lee[2,3], A. Ozgul[1], D. R. Farine,[1,4], and B. König[1],

[1]Department of Evolutionary Biology and Environmental Studies, University of Zurich, Zurich, Switzerland

[2]Wild Nature Institute, Concord, New Hampshire, USA

[3]Department of Biology, Pennsylvania State University, University Park, Pennsylvania, USA

[4]Department of Collective Behavior, Max Planck Institute of Animal Behavior, Konstanz, Germany

[*]**Corresponding author**. Email: monica.bond@ieu.uzh.ch

Supplementary Information

Appendix 1: Tarangire Ecosystem

Vegetation types ranged from open grasslands to dense deciduous bushlands and thickets (Lamprey, 1963). We sampled within a 2200 km^2 area along road transects in four administrative areas with differing management activities (*Fig. S1*). Land management was partitioned among Tarangire National Park, Manyara Ranch Conservancy, and Mto wa mbu and Lolkisale Game Controlled Areas. Tarangire National Park had high levels of wildlife protection including exclusion of livestock and human settlements and rigorous anti-poaching patrols; Manyara Ranch had intermediate levels of wildlife protection with exclusion of human settlements and some anti-poaching patrols but with large numbers of livestock during the daytime. Portions of Lolkisale and Mto wa mbu Game Controlled Areas adjacent to Tarangire National Park had village Wildlife Management Areas with anti-poaching efforts similar to Manyara Ranch and exclusion of permanent human settlements, but the remainder of the region had sport hunting and permanent human settlements—although hunting of giraffes is prohibited. All administrative areas were unfenced and connected by movements of adult female giraffes (Lee & Bolger, 2017).

The Rift Valley escarpment, a steep cliff that restricts giraffe movements, formed the western boundary of the study area. Wild large mammals are rarely observed east of Lolkisale Game Controlled Area and Manyara Ranch due to high human and livestock population densities, or southwest of Tarangire National Park due to widespread intensive agriculture. Two 2-lane asphalt roads crossed the study area.

Appendix 2: Social network analysis and community detection

For social network analysis and constructing social communities to examine social dispersal, we included the calf dataset detailed in the main text as well as all females first observed during the

initial year of the study that (1) were adults (≥4 yr) the first time they were observed based on visual determination in the field, and (2) were detected ≥6 times over the course of the study to improve accuracy of social network analysis (Davis et al., 2018). This resulted in a subset of 423 adult females from a total of 1039 identified during the entire study period. We used the gambit of the group to define associations, whereby individuals present together in the same group formation were considered associated during that survey (Whitehead & Dufault, 1999). We defined a group formation as one or more giraffes that were foraging or moving together, and with >500 m distance to the closest member of another group (Carter, Seddon, et al., 2013; Carter, Brand, et al., 2013; VanderWaal et al., 2014, Bond et al., 2019). Our study design involved repeatedly measuring individually identified giraffes, with each individual assigned to one group formation per secondary sampling period.

We created a social network of adult females over the 7-year study period from the simple ratio index of association (Farine & Whitehead, 2015) using package *asnipe* (Farine, 2013) for R version 3.5.3 (R Core Development Team, 2019). We ran community detection algorithms on the network with package *igraph* (Csárdi & Nepusz, 2006) and tested robustness of our community detection using the approach and test statistic r_{com}, described by Shizuka and Farine (2016). We calculated r_{com} with package *assortnet* for R (Farine, 2016).

We ran four community detection algorithms on the network of adult females. The highest modularity Q was the cluster-walktrap (0.689), followed by cluster fast greedy (0.667), cluster edge betweenness (0.661), and leading eigenvector (0.645). The cluster-walktrap algorithm parsed the adult females into 12 social communities (*Fig. S1a*), and the bootstrap test revealed that our community assignment was robust (r_{com} = 0.765).

We tested temporal stability of adult female communities by comparing community assignment in a network generated from detections in 2012–2014, with a network generated from

detections in 2016–2018, using a subset of 373 females seen ≥6 times during each of the two time periods. Every adult female was placed in a community with the same adult females at both the beginning and end of the study period. The highly stable communities we observed over our 7-year time frame align with previous research in Namibia showing constant relationships among pairs of adult female giraffes over 6 years (Carter, Brand, et al., 2013).

Appendix 3: Estimating age with photogrammetry

For each detection in our dataset, we estimated size as a proxy for age with photogrammetry (Schrader et al., 2006). We calculated multiple heights for each individual to confirm our age estimates. We used data from known-age calves born in captivity to estimate age-specific neck length (NL), total height (TH), and the ratio of NL/TH for giraffes aged 0 to 6 months (M. L. Bond, unpublished data). These data and other studies (Dagg, 2014) found no differences in height between male and female giraffe calves during the first two years of life. For images of our wild giraffes, we measured the number of pixels along the length of the giraffe's neck on all photographs. We used algorithms incorporating the focal length of the lens and distance to the subject to convert pixels to cm and estimate NL and thus age when the photograph was taken. For additional details on photogrammetry methods for giraffes, see *Supporting Information S4* in Lee et al. (2016).

Appendix 4: Estimating socio-ecological correlates of dispersal

We used all the group locations of all adult female community members to estimate a 95% kernel home range for the community using the package *adehabitatHR* in R (Calenge, 2006). The number of group locations for estimating community home ranges ranged from 23 to 269 (mean = 153.6 locations). Börger et al. (2006) showed that if the sampling regime is standardized (as in our case, regular standardized surveys throughout the study area during the entire study

period), then inferences should be robust to sampling variation in the number of locations. We conducted a simple linear regression to determine whether home range size was correlated with the number of group locations among communities, and found no significant correlation (($F_{1,10}$ = 0.116, p = 0.74). We consider the estimated home ranges to adequately reflect the approximate use of space by the community over the 7-year study period; we used the home ranges to estimate the approximate amount of food resources available to them and the local giraffe population density during this time. We utilized ground-based measurements that we collected in January 2014 every 2 km along our survey transects to map polygons of vegetation with >10% cover of one of three primary giraffe forage species: (1) *Vachelia tortilis*; (2) *V. drepanolobium*; and (3) *Dichrostachys cinerea*. Based on foraging selection analyses, our population of giraffes in the Tarangire Ecosystem strongly selected these three plants during both wet and dry seasons, and the three species together comprised approximately 60% of the giraffes' diet (Levi et al., in press).

To test for anthropogenic influences on dispersal, we computed the average distance (km) from all locations of community members to the nearest boma (a low-impact human settlement) and the nearest town (a high-impact human settlement). We used Google Earth imagery to map all bomas and towns, and to compute distances we used the "Generate Near Table" function in the Analysis Tools toolbox in ArcMap™ version 10.8.1 (Esri, Redlands, CA, USA). Finally, we calculated the community's local giraffe population density, which included all individually identified adult male and female giraffes ever detected in each community's home range (including overlapping communities), regardless of their number of detections.

We tested for correlations among the socio-ecological covariates and did not use two variables together in the same model if they were highly correlated (Table S1). Models tested and model selection results are presented in Table S2.

Table S1. Spearman rank correlations for socio-ecological covariates to dispersal for 137 female and male Masai giraffe calves in 12 adult female social communities in the Tarangire Ecosystem of northern Tanzania, 2012–2018. Bold indicates correlation coefficient > 0.50.

	Population density[1]	Distance to town[2]	Distance to boma[3]	Proportion *V. drepanolobium*[4]	Proportion *V. tortilis*[5]
Distance to town	**-0.63**				
Distance to boma	-0.20	0.30			
Proportion *V. drepanolobium*	**0.73**	**-0.69**	0.28		
Proportion *V. tortilis*	-0.21	**0.53**	-0.41	**-0.68**	
Proportion *D. cinerea*[6]	0.42	**-0.77**	-0.26	0.41	**-0.55**

[1] Local giraffe population density in each social community of adult females, included all individual identified adult male and female giraffes ever detected in each community's home range.
[2] Mean distance (in km) from all locations of social community members to the nearest town.
[3] Mean distance (in km) from all locations of social community members to the nearest boma.
[4] Proportion of *Vachellia drepanolobium* in the social community's home range.
[5] Proportion of *Vachellia tortilis* in the social community's home range.
[6] Proportion of *Dichrostachys cinerea* in the social community's home range.

Table S2. Model selection results ranking 20 linear regression models explaining variation in dispersal type (no dispersal, social only, spatial only, social-and-spatial); minimal dispersal distance, defined as the Euclidean distance (km) from first (natal) to last location; and age of dispersal (months) for male and female giraffe calves in the Tarangire Ecosystem of northern Tanzania, 2012–2018, based on maximum likelihood estimation.

**Dispersal Type Models**	**d.f.**	**LogLik**	**ΔAICc**	**Weight**
All animals				
Sex + DistTown[1]	9	-137.67	0.00	0.53
Sex + *V. drepanolobium*[2]	9	-138.90	2.47	0.15
Sex + DistBoma[3]	9	-139.40	3.46	0.09
Sex + *D. cinerea*[4]	9	-139.77	4.20	0.06
Sex + DistBoma + PopDens[5]	12	-136.78	4.22	0.06
Sex + PopDens	9	-140.12	4.90	0.05
Sex + PopDens + *D. cinerea*	12	-137.82	6.30	0.02
Sex + PopDens + *V. drepanolobium*	12	-138.65	7.96	0.01
Sex	6	-145.18	9.01	0.01
Sex + DistBoma + PopDens + (DistBoma × PopDens)	15	-136.48	9.62	0.00
Sex + DistBoma + *V. drepanolobium* + (Sex × DistBoma) + (Sex × *V. drepanolobium*)	18	-134.53	11.72	0.00
Sex + DistBoma + *D. cinerea* + (Sex × DistBoma) + (Sex × *D. cinerea*)	18	-134.82	12.30	0.00
Sex + *V. tortilis*[6]	9	-144.90	14.46	0.00
Sex + DistBoma + *V. tortilis* + (Sex × DistBoma) + (Sex × *V. tortilis*)	18	-137.11	16.87	0.00
DistTown	6	-154.06	26.78	0.00
DistBoma	6	-156.01	30.69	0.00
V. drepanolobium	6	-156.61	31.87	0.00

D. cinerea	6	-157.07	32.80	0.00
Null	3	-161.85	36.37	0.00
V. tortilis	6	-161.61	41.89	0.00
**Dispersal Distance Models**	**d.f.**	**LogLik**	**ΔAICc**	**Weight**
Sex + DistTown	4	-444.79	0.00	0.86
Sex + *V. drepanolobium*	4	-447.58	5.57	0.05
Sex + DistBoma + PopDens + (DistBoma × PopDens)	6	-446.32	7.40	0.02
Sex + PopDens + *V. drepanolobium*	5	-447.50	7.57	0.02
Sex + DistBoma + *V. drepanolobium* + (Sex × DistBoma) + (Sex × *V. drepanolobium*)	7	-445.44	7.86	0.02
DistTown	3	-449.81	7.92	0.02
Sex + PopDens + *D. cinerea*	5	-448.27	9.11	0.01
Sex + PopDens	4	-450.33	11.08	0.00
Sex + *D. cinerea*	4	-450.68	11.76	0.00
Sex + DistBoma + PopDens	5	-449.81	12.18	0.00
Sex + DistBoma + *D. cinerea* + (Sex × DistBoma) + (Sex × *D. cinerea*)	7	-449.90	16.78	0.00
Sex + DistBoma	4	-453.49	17.38	0.00
V. drepanolobium	3	-455.04	18.37	0.00
Sex	3	-455.50	19.29	0.00
Sex + *V. tortilis*	4	-455.08	20.57	0.00
Sex + DistBoma + *V. tortilis* + (Sex × DistBoma) + (Sex × *V. tortilis*)	7	-452.28	21.55	0.00
D. cinerea	3	-456.82	21.93	0.00
DistBoma	3	-458.81	25.91	0.00
Null	2	-460.88	27.96	0.00
V. tortilis	3	-460.66	29.61	0.00
**Dispersal Age Models**	**d.f.**	**LogLik**	**ΔAICc**	**Weight**

DistTown	3	-213.04	0.00	0.33
Sex + DistTown	4	-212.93	2.15	0.11
V. drepanolobium	3	-214.20	2.32	0.10
DistBoma	3	-214.24	2.40	0.10
Null	2	-215.44	2.53	0.09
V. tortilis	3	-214.99	3.90	0.05
D. cinerea	3	-215.31	4.53	0.03
Sex	3	-215.36	4.63	0.03
Sex + DistBoma	4	-214.19	4.65	0.03
Sex + *V. drepanolobium*	4	-214.19	4.65	0.03
Sex + PopDens	4	-214.59	5.46	0.02
Sex + *V. tortilis*	4	-214.88	6.03	0.02
Sex + DistBoma + PopDens	5	-213.82	6.39	0.01
Sex + *D. cinerea*	4	-215.26	6.80	0.01
Sex + PopDens + *V. drepanolobium*	5	-214.17	7.10	0.01
Sex + DistBoma + PopDens + (DistBoma × PopDens)	6	-213.29	7.92	0.01
Sex + PopDens + *D. cinerea*	5	-214.59	7.94	0.01
Sex + DistBoma + *V. tortilis* + (Sex × DistBoma) + (Sex × *V. tortilis*)	7	-212.13	8.32	0.01
Sex + DistBoma + *D. cinerea* + (Sex × DistBoma) + (Sex × *D. cinerea*)	7	-213.07	10.19	0.00
Sex + DistBoma + *V. drepanolobium* + (Sex × DistBoma) + (Sex × *V. drepanolobium*)	7	-213.27	10.60	0.00

[1] DistTown = mean distance (km) from all locations of community members to the nearest high-impact human settlement.

[2] Proportion of *Vachellia drepanolobium* in the social community's home range.

[3] DistBoma= mean distance (km) from all locations of community members to the nearest low-impact human settlement.

[4] Proportion of *Dichrostachys cinerea* in the community's home range.

[5] PopDens = all individually identified adult giraffes ever detected in the community's kernel home range.

[6] Proportion of *Vachellia tortilis* in the community's home range

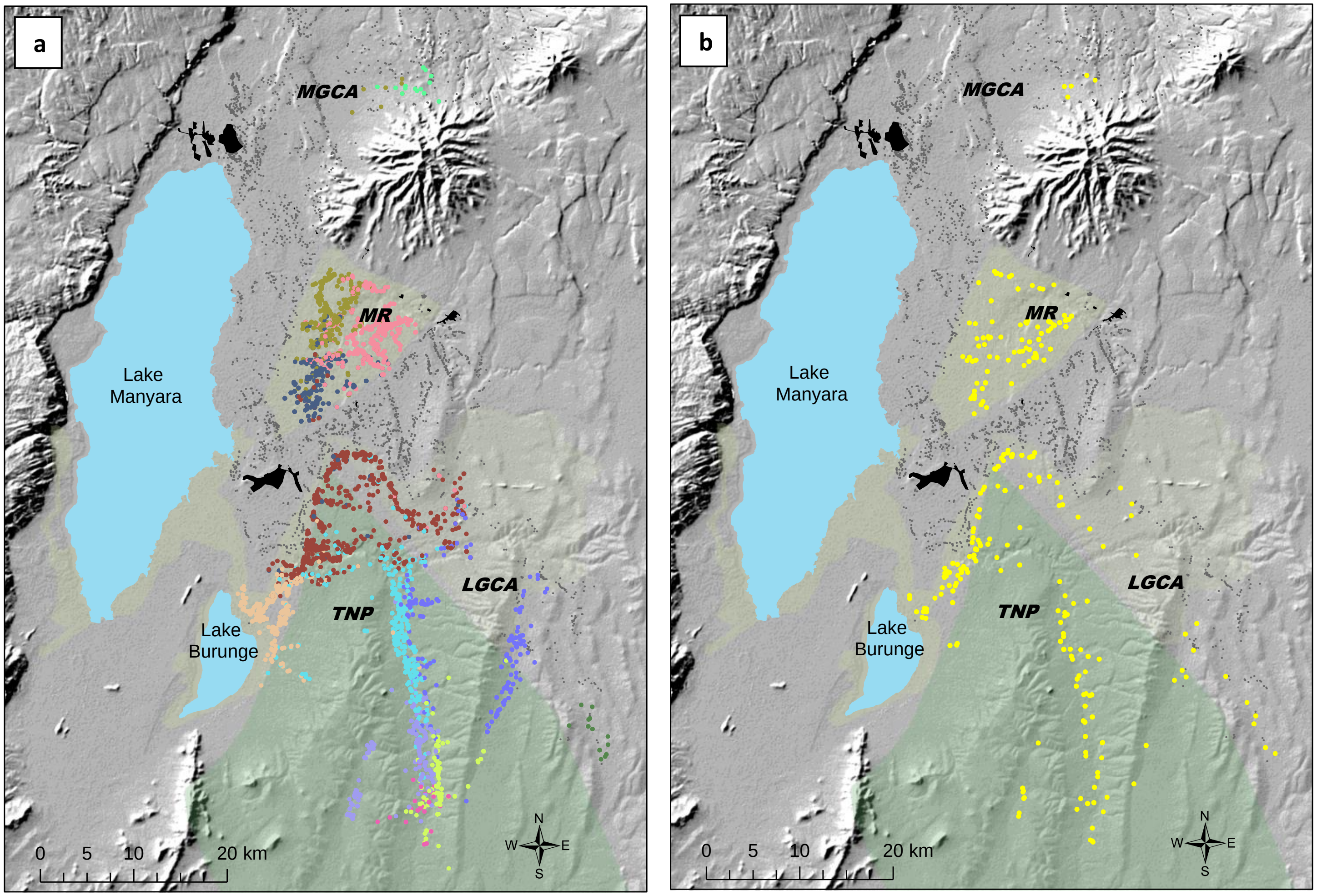

Figure S1. (a) Locations (colored points) of adult female Masai giraffes in 12 communities in the Tarangire Ecosystem, Tanzania, from 2012–2018. TNP is Tarangire National Park, MR is Manyara Ranch Conservancy, MGCA is Mto wa mbu Game Controlled Area, and LCGA is Lolkisale Game Controlled Area. Green polygons are protected areas TNP, MR, and two community Wildlife Management Areas. Grey points are bomas, and black polygons are towns. **(b)** Locations (yellow points) of first detection of 137 giraffe calves used in dispersal analysis.

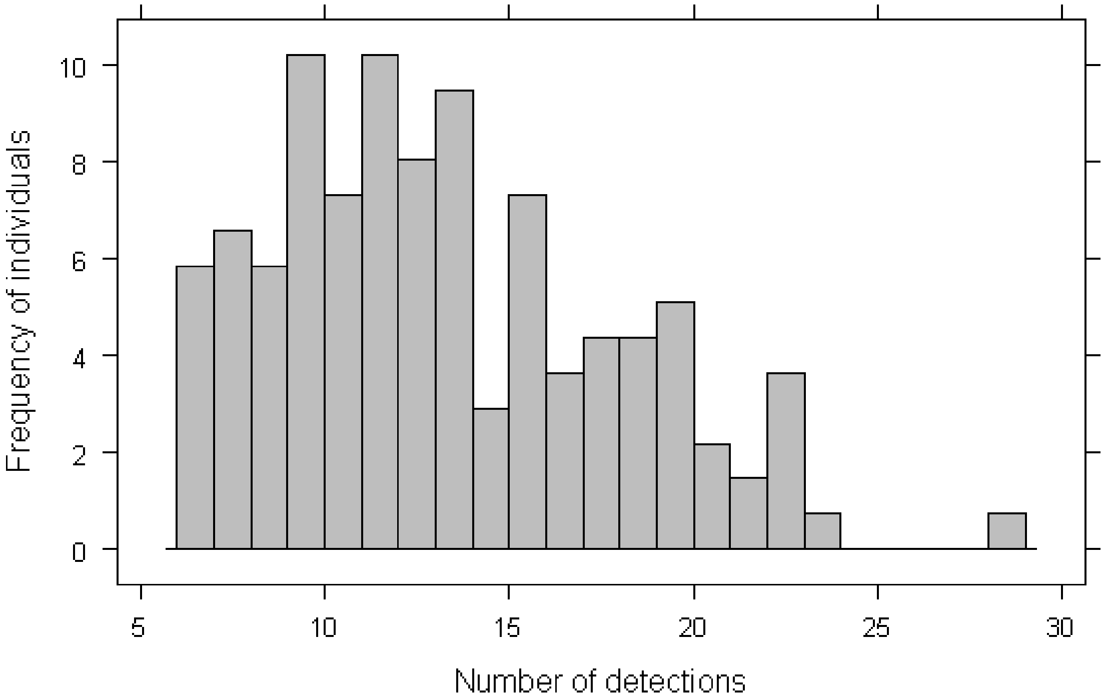

Figure S2. Range of number of detections for 137 Masai giraffe calves (67 M and 70 F) used in the dispersal analysis in the Tarangire Ecosystem, Tanzania, from 2012–2018.

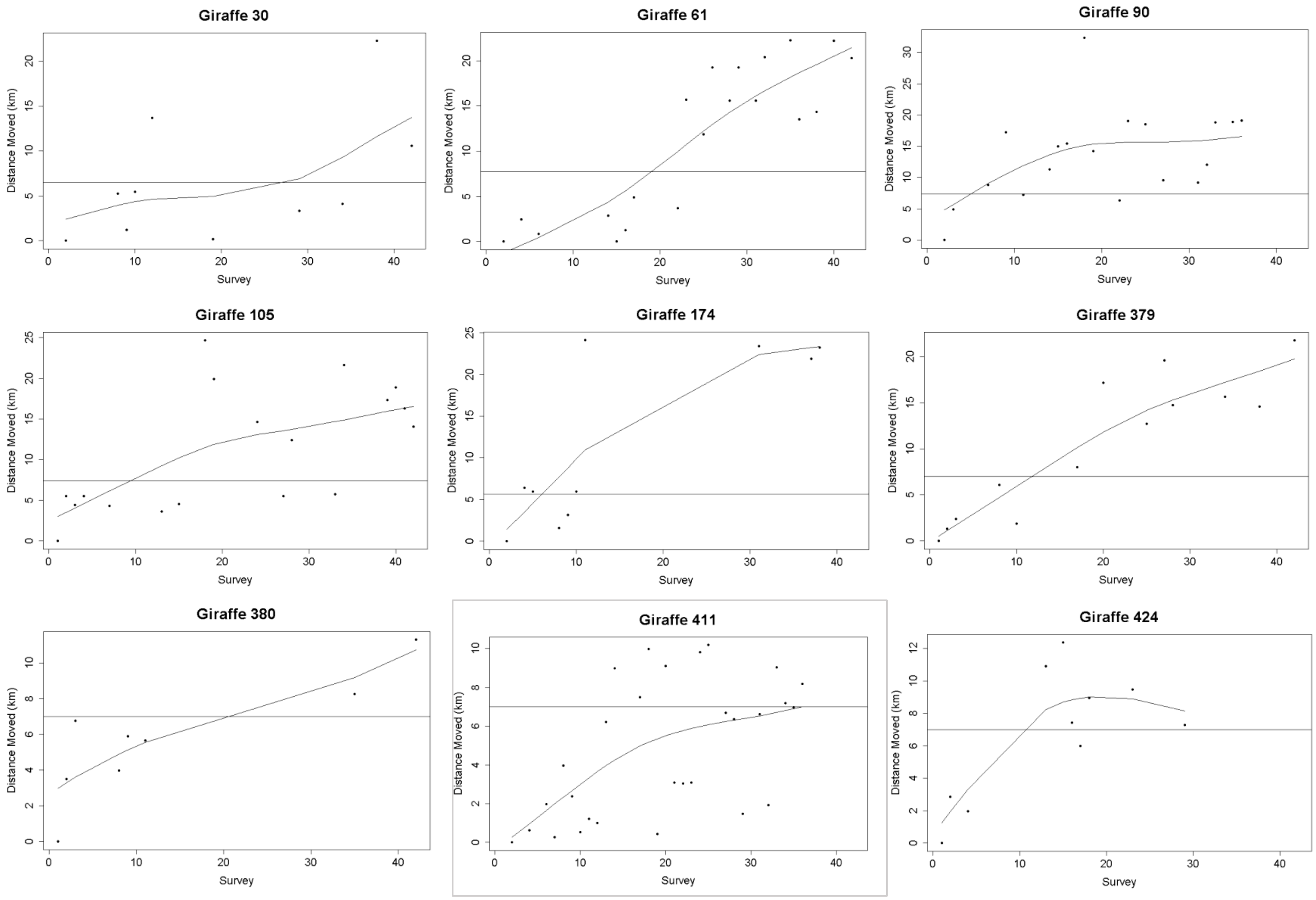

Giraffe 30
Giraffe 61
Giraffe 90
Giraffe 105
Giraffe 174
Giraffe 379
Giraffe 380
Giraffe 411
Giraffe 424
Distance Moved (km)
Survey

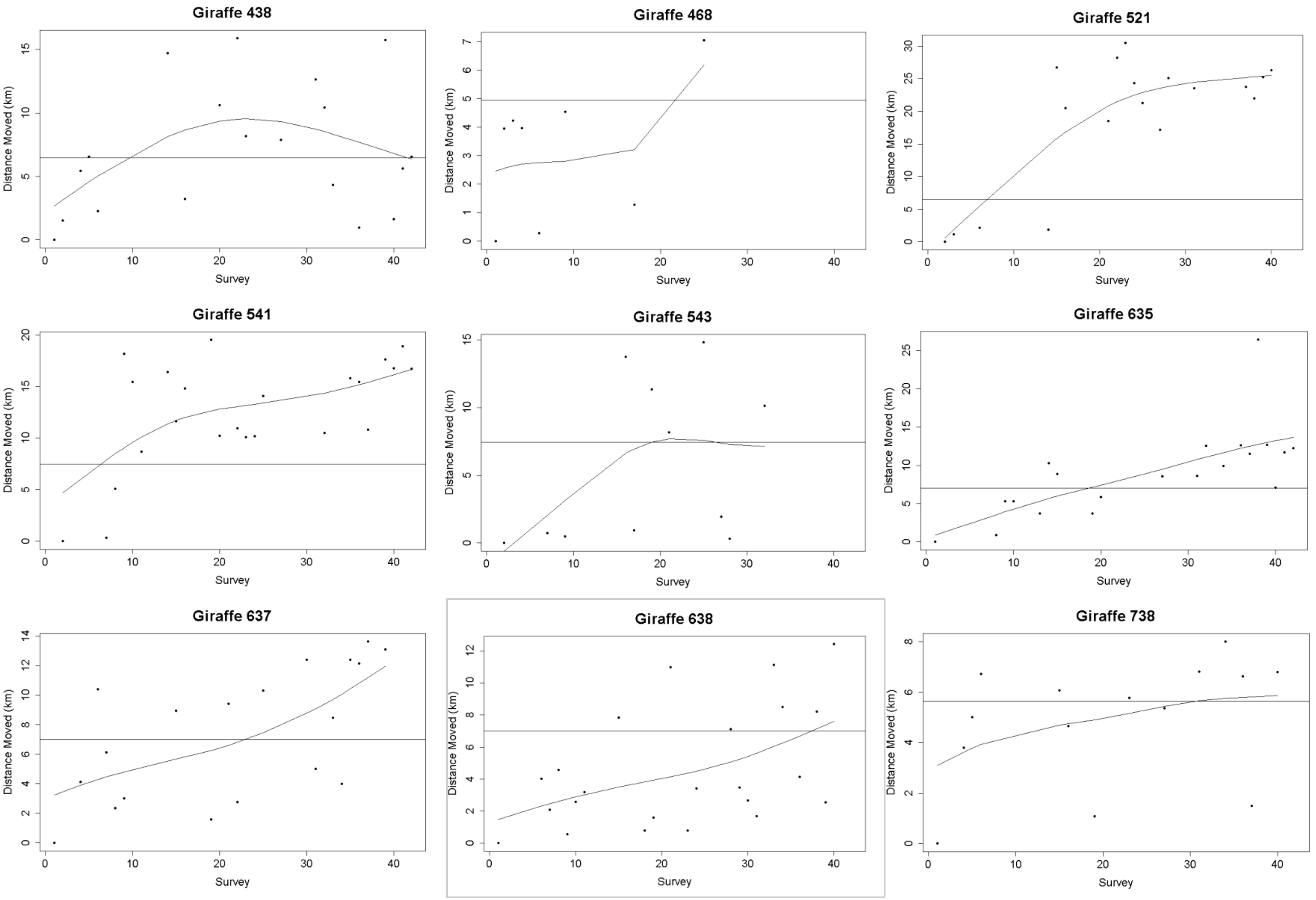
Giraffe 438
Giraffe 468
Giraffe 521
Giraffe 541
Giraffe 543
Giraffe 635
Giraffe 637
Giraffe 638
Giraffe 738
Distance Moved (km)
Survey

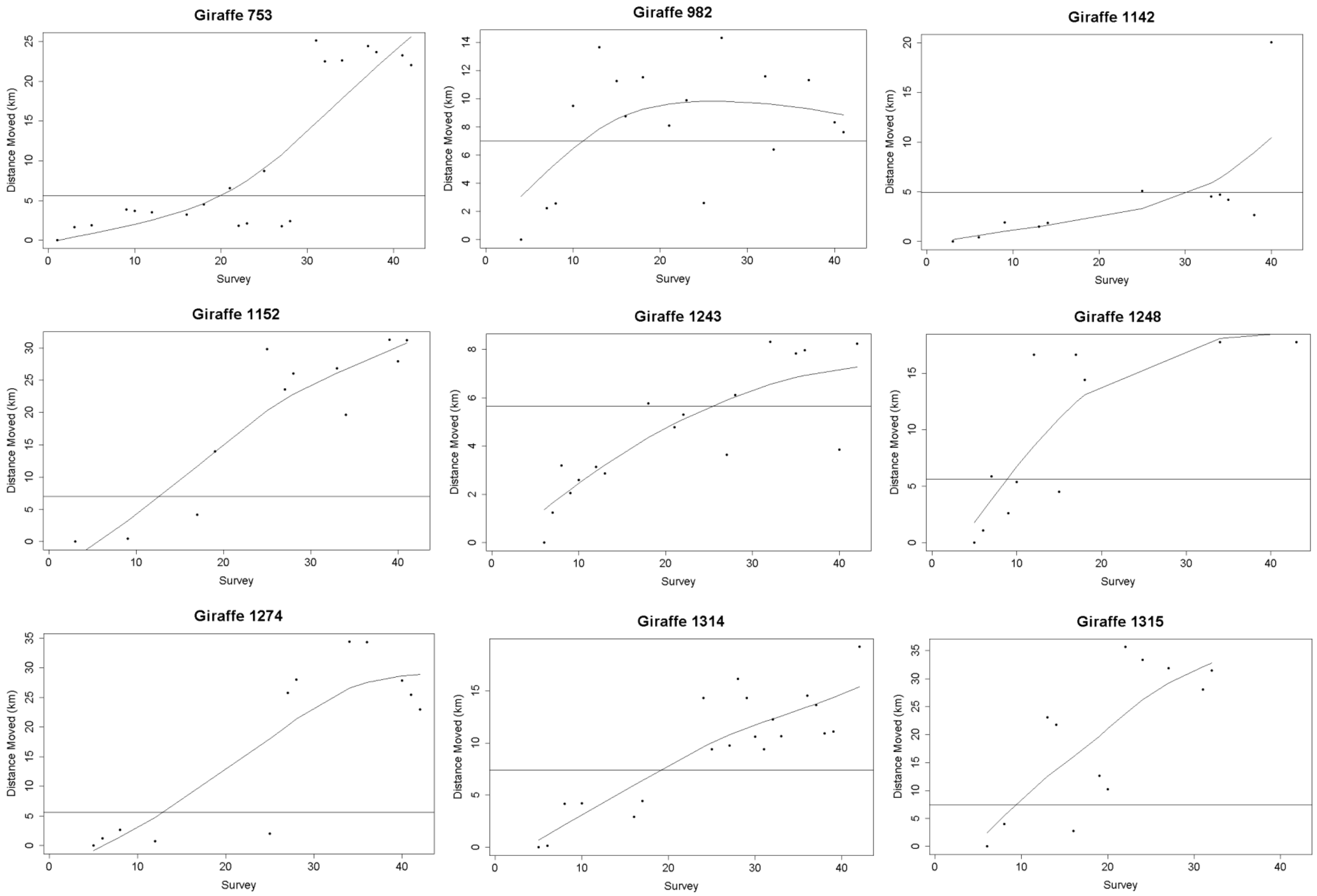
Giraffe 753
Giraffe 982
Giraffe 1142
Giraffe 1152
Giraffe 1243
Giraffe 1248
Giraffe 1274
Giraffe 1314
Giraffe 1315
Distance Moved (km)
Survey

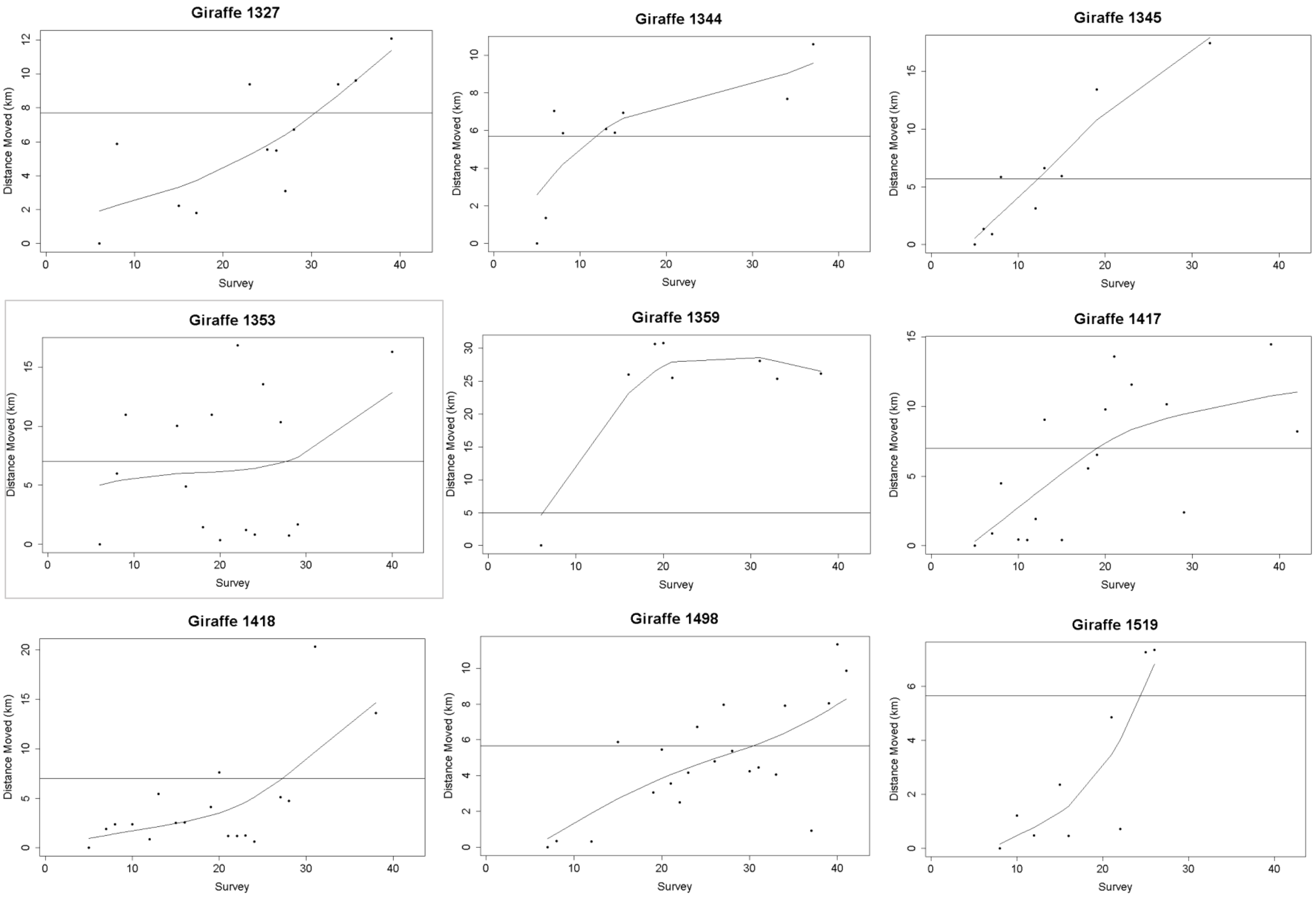
Giraffe 1327
Giraffe 1344
Giraffe 1345
Giraffe 1353
Giraffe 1359
Giraffe 1417
Giraffe 1418
Giraffe 1498
Giraffe 1519
Distance Moved (km)
Survey

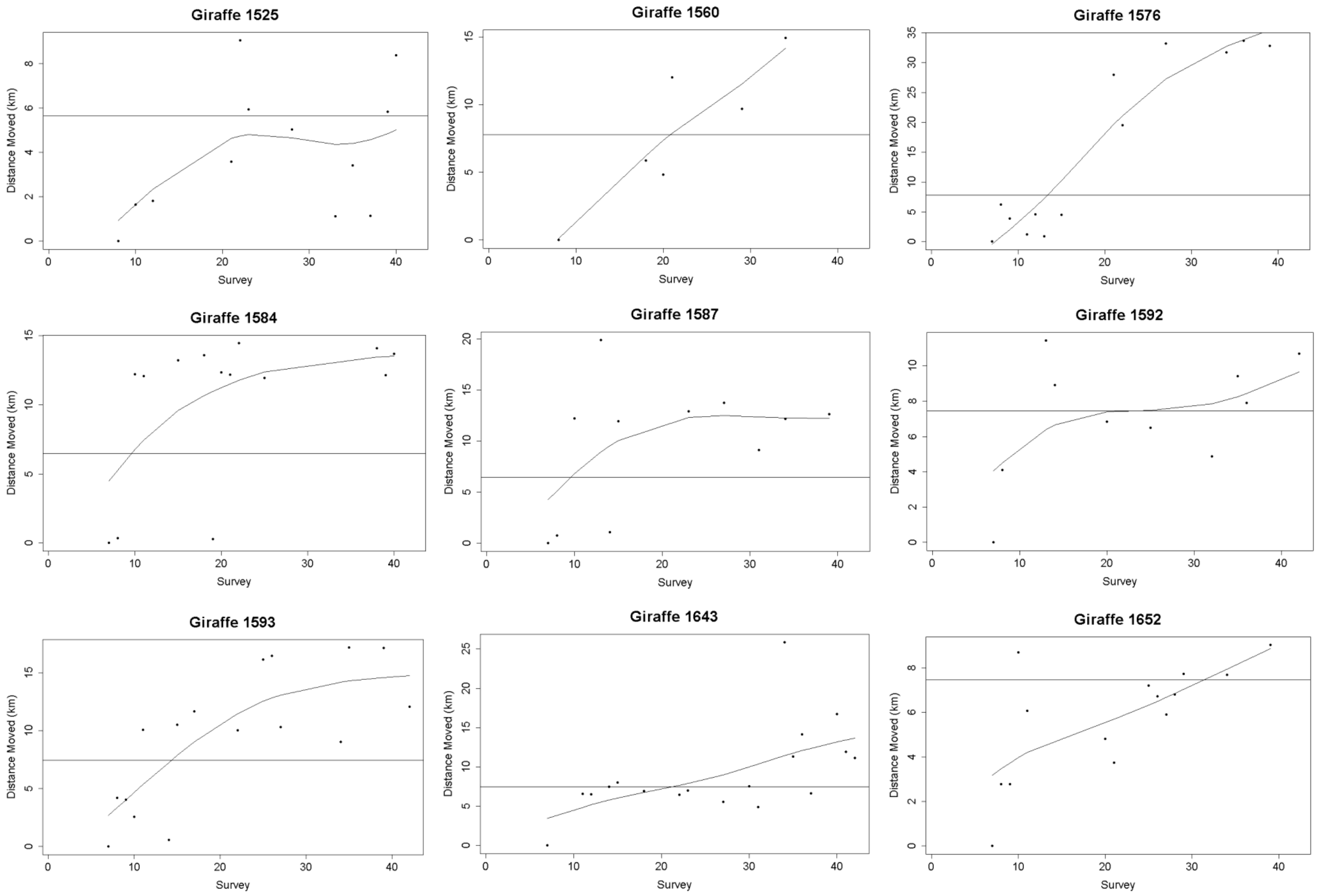
Giraffe 1525
Giraffe 1560
Giraffe 1576
Giraffe 1584
Giraffe 1587
Giraffe 1592
Giraffe 1593
Giraffe 1643
Giraffe 1652
Distance Moved (km)
Survey

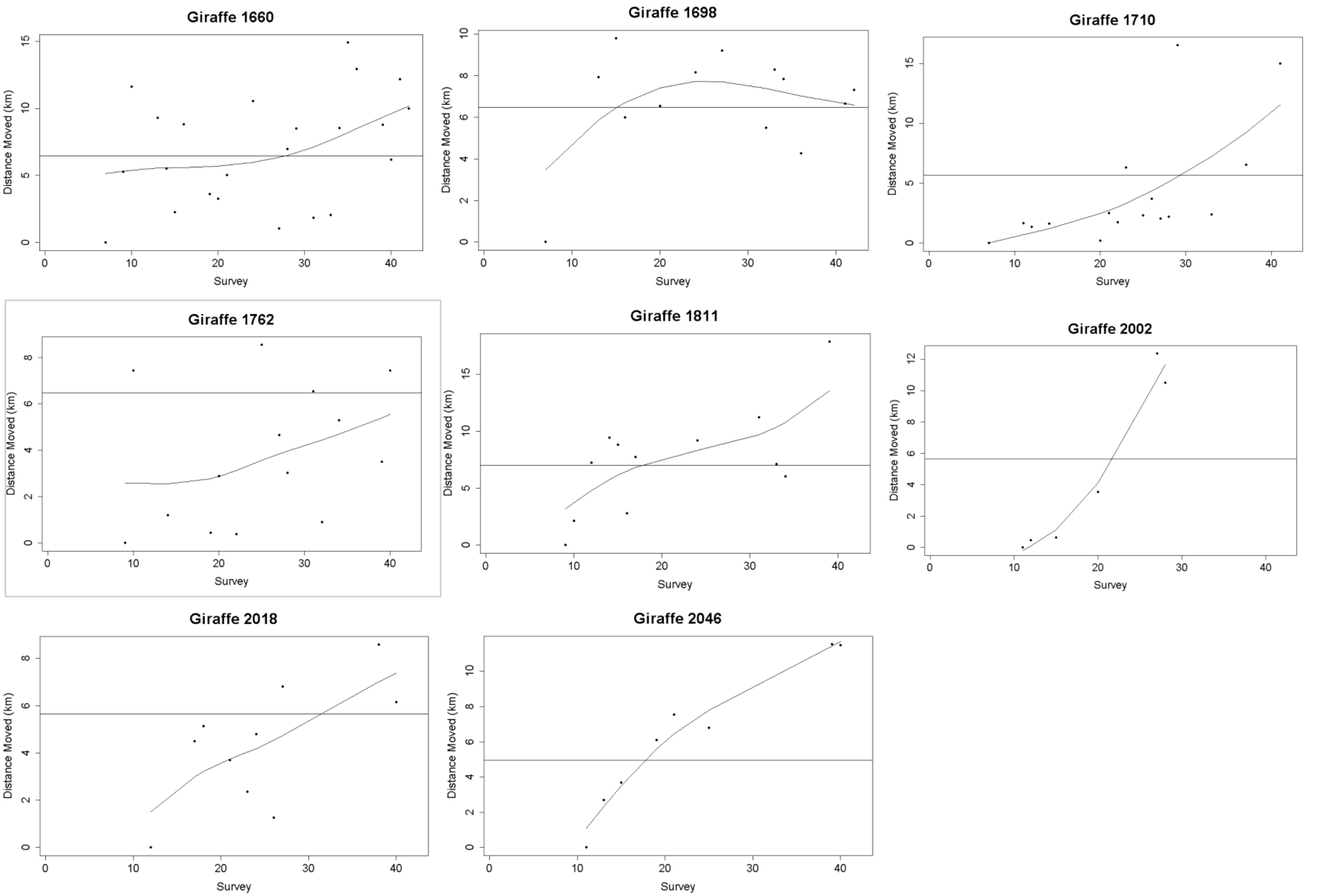

Giraffe 1660
Giraffe 1698
Giraffe 1710
Giraffe 1762
Giraffe 1811
Giraffe 2002
Giraffe 2018
Giraffe 2046
Distance Moved (km)
Survey

Figure S3. Survey-specific distance from origin of 53 male and female Masai giraffe calves whose final detection exceeded the radius of an average adult female home range in the calf's natal social community. The x-axis represents the survey number (N = 42), the y-axis is distance (km), and the horizontal line represents the home range radius. Each point represents a detection of the individual and the Euclidian distance of that point from its first detection (first detection = 0 km). Individuals were classified as spatial dispersers if, after a smoothed line fitted through all the points exceeded the radius threshold, more points were beyond the threshold. The figures with grey squares are four females removed as spatial dispersers and re-classified as non-dispersers that made spatial sorties.

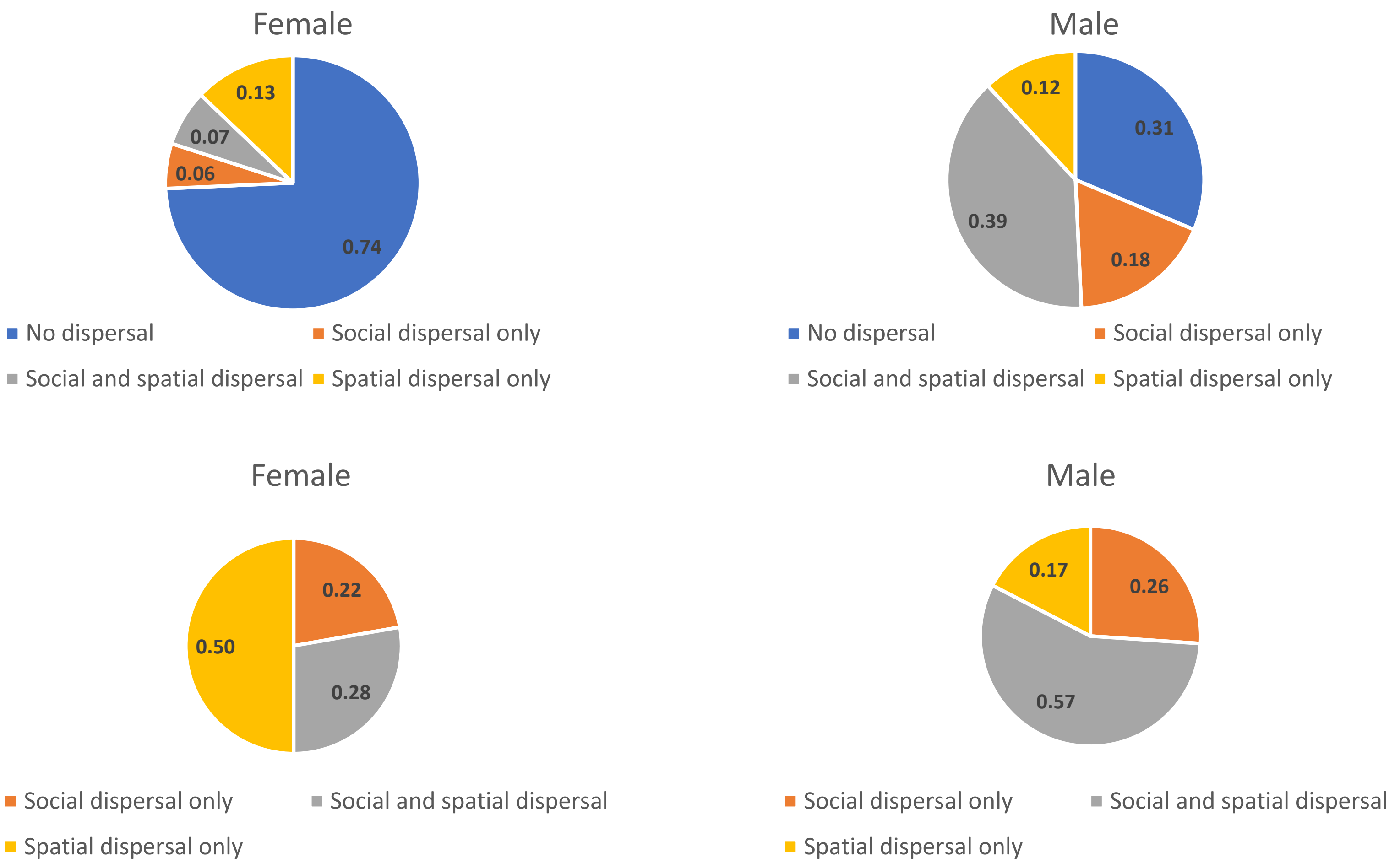

Figure S4. Proportion of dispersers by type (no dispersal, social dispersal only, spatial dispersal only, social-and-spatial dispersal) for all female and male Masai giraffe calves, and for female and male dispersers, in the Tarangire Ecosystem, Tanzania, 2012–2018.

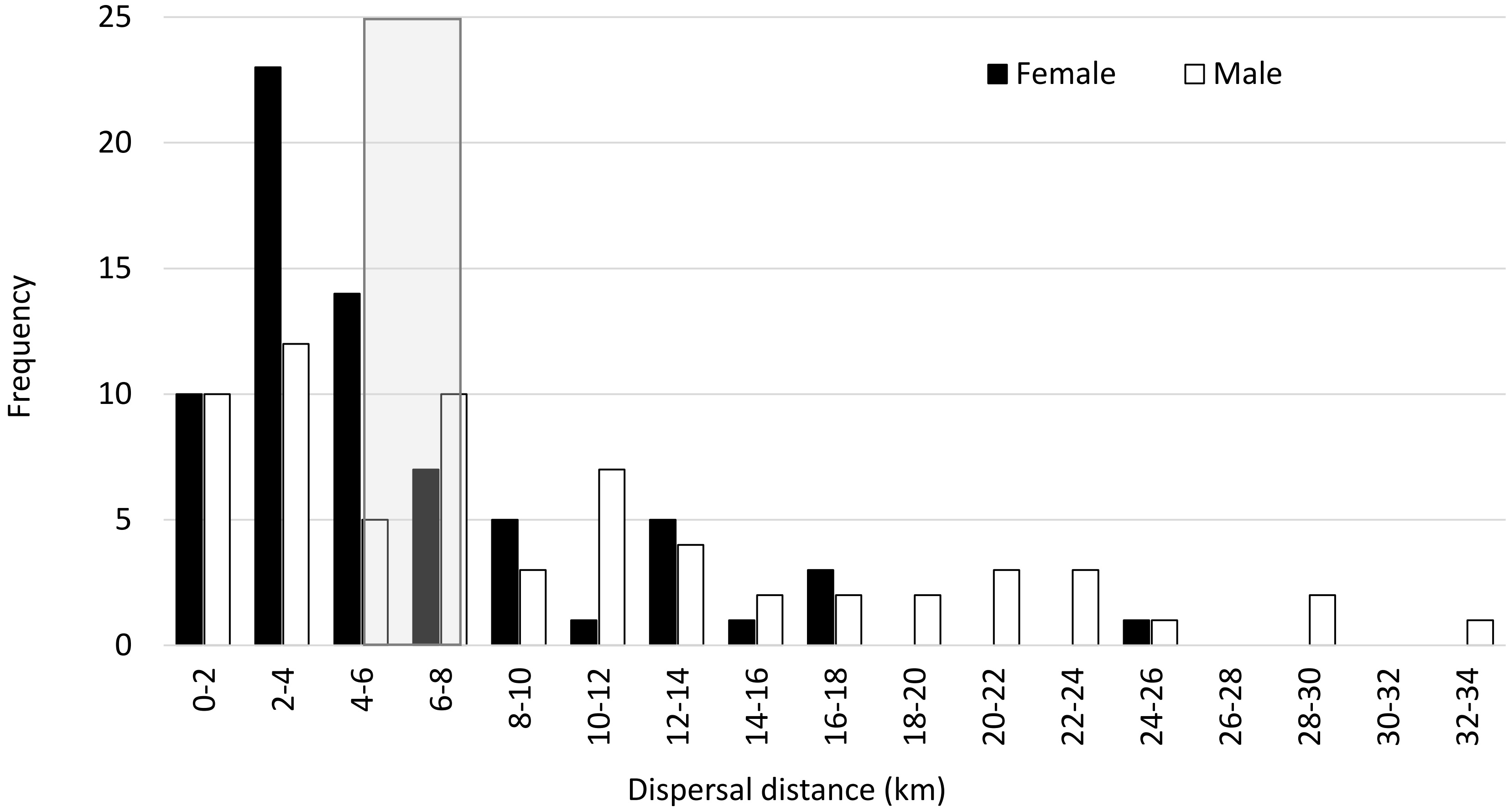

Figure S5. Histogram of dispersal distance (Euclidean distance moved from first detection as a calf to last detection as subadult) in kilometers by frequency of individuals, for 137 male and female Masai giraffes in the Tarangire Ecosystem, Tanzania, from 2012–2018. Open bar represents range of threshold dispersal distances for spatial dispersal (4.95 – 7.77 km), which varied by adult female social community. The threshold distance was the radius of an average adult female home range size in the calf's natal community.

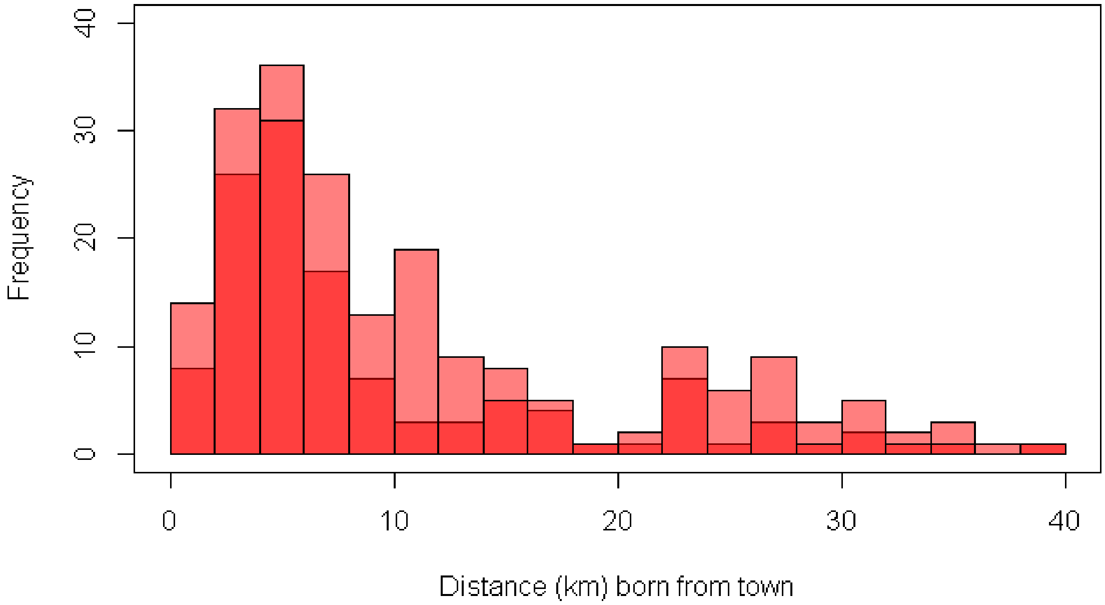

Figure S6. Frequency of individual Masai giraffe calves first sighted at different distances from towns (km) in the Tarangire Ecosystem, Tanzania, 2012–2018. Pink = number of individuals included in dispersal dataset (*n* = 137), red = not included (*n* = 191). The distance from towns did not differ between the two samples (Wilcoxon rank sum test with continuity correction, *W* = 14008, *P* = 0.28).

References

Bond, M.L., Lee, D.E., Ozgul, A., & König, B. (2019). Fission-fusion dynamics of a megaherbivore are driven by ecological, anthropogenic, temporal, and social factors. *Oecologia*, 191, 335–347.

Börger, L., Franconi, N., De Michele, G., Gantz, A., Meschi, F., Manica, A., Lovari, S., & Coulson, T. (2006). Effects of sampling regime on the mean and variance of home range size estimates. *Journal of Animal Ecology* 75, 1393–1405.

Calenge, C. (2006). The package adehabitat for the R software: a tool for the analysis of space and habitat use by animals. *Ecological Modelling* 197, 516–519.

Carter, K.D., Seddon, J.M., Frère, C.H., Carter, J.K., & Goldizen, A.W. (2013a). Fission-fusion dynamics in wild giraffes may be driven by kinship, spatial overlap and individual social preferences. *Animal Behaviour* 85, 385–394.

Carter, K.D., Brand, R., Carter, J.K., Shorrocks, B., & Goldizen, A.W. (2013b). Social networks, long-term associations and age-related sociability of wild giraffes. *Animal Behaviour* 86, 901–910.

Csárdi G., & Nepusz T. (2006). The igraph software package for complex network research. InterJournal for Complex Systems 1695.

Dagg, A.I. (1971). *Giraffa camelopardalis*. *Mammalian Species* 5, 1–8.

Dagg, A.I. (2014). Giraffe Biology, Behavior and Conservation. Cambridge University Press, New York, New York, USA.

Davis, G.H., Crofoot, M.C., & Farine, D.R. (2018). Estimating the robustness and uncertainty of animal social networks using different observational methods. *Animal Behaviour* 141, 29–44.

Farine, D.R. (2013). Animal social network inference and permutations for ecologists in R using asnipe. *Methods in Ecology and Evolution* 4, 1187–1194.

Farine, D.R. (2016). assortnet: Calculate the Assortativity Coefficient of Weighted and Binary Networks. R package version 0.12. https://CRAN.R-project.org/package=assortnet

Farine, D.R., & Whitehead, H. (2015). Constructing, conducting and interpreting animal social network analysis. *Journal of Animal Ecology* 84, 1144–1163.

Lamprey, H.F. (1963). The Tarangire Game Reserve. *Tanganyika Notes and Records* 60, 10–22.

Lee, D.E., & Bolger, D.T. (2017). Movements and source-sink dynamics of a Masai giraffe metapopulation. *Population Ecology* 59, 157–168.

Lee, D.E., Bond, M.L., Kissui, B.M., Kiwango, Y.A., & Bolger, D.T. (2016). Spatial variation in giraffe demography: a test of 2 paradigms. *Journal of Mammalogy* 97, 1015–1025.

Levi, M., Lee, D.E., Bond, M.L., & Treydte, A.C. (in press). Forage selection by Masai giraffes (*Giraffa camelopardalis tippelskirchi*) at multiple spatial scales. *Journal of Mammalogy*.

R Core Development Team (2019). R: a language and environment for statistical computing. R Foundation for Statistical Computing, Vienna, Austria.

Schrader, A.M., Ferreira, S.M., & van Aarde, R.J. (2006). Digital photogrammetry and laser rangefinder techniques to measure African elephants. *South African Journal of Wildlife Research* 36, 1–7.

Shizuka, D., & Farine, D.R. (2016). Measuring the robustness of network community structure using assortativity. *Animal Behaviour* 112, 237–246.

VanderWaal, K.L., Wang, H., McCowan, B., Fushing, H., & Isbell, L.A. (2014). Multilevel social organization and space use in reticulated giraffe (*Giraffa camelopardalis*). *Behavioral Ecology* 25, 17–26.

Whitehead, H., & Dufault, S. (1999). Techniques for analysing vertebrate social structure using identified individuals: review and recommendations. *Advances in the Study of Behavior* 28, 33–74.
